# Prognostic value of Lin28A and Lin28B in various human malignancies: a systematic review and meta-analysis

**DOI:** 10.1186/s12935-019-0788-z

**Published:** 2019-04-02

**Authors:** Jiayi Zhang, Aiming Xu, Chenkui Miao, Jie Yang, Min Gu, Ninghong Song

**Affiliations:** Department of Urology, The First Affiliated Hospital of Nanjing Medical University, Nanjing, 210029 China

**Keywords:** Lin-28, Prognosis, Human tumors, Biomarker, Meta-analysis

## Abstract

**Background:**

The mammalian homologs of Lin-28, Lin28 (also called Lin28A) and Lin28B, are promising cancer biomarkers. This meta-analysis was performed to evaluate the prognostic values of Lin28A and Lin28B in multiple human malignancies.

**Methods:**

Systematic searches of the PubMed, Web of Science and Embase were used to identify relevant studies. Pooled hazard ratios (HRs) with 95% confidence intervals (CI) for overall survival (OS), recurrence-free survival (RFS), disease-free survival (DFS), or progression-free survival (PFS) were respectively calculated.

**Results:**

3772 Lin28A-associated patients and 1730 Lin28B-related cases were ultimately enrolled in this meta-analysis. The elevated expression level of Lin28A was significantly associated with poor OS (HR = 1.60, P < 0.001) and poor RFS/DFS/PFS (HR = 1.62, P < 0.001) in patients with malignancies. Lin28B overexpression significantly correlated with unfavorable OS (HR = 1.72, P < 0.001) and RFS/DFS/PFS (HR = 2.35, P < 0.001) of human malignancies.

**Conclusions:**

Lin28A and Lin28B possess significant prognostic values in various human malignancies. Overexpression of Lin28A or Lin28B suggests poor prognosis for cancer patients.

**Electronic supplementary material:**

The online version of this article (10.1186/s12935-019-0788-z) contains supplementary material, which is available to authorized users.

## Background

In recent years, cancer has become the primary cause of mortality in most countries and regions, and the incidence of human malignancies has increased substantially [1]. Given the low survival rate of multiple cancer types, credible biomarkers for cancer prognosis are urgently required. Recently, the mammalian homologs of Lin-28, Lin28 (also called Lin28A) and Lin28B, have been considered as promising biomarkers.

Lin28A and Lin28B are highly conserved RNA-binding and microRNA-regulated proteins. In general, they selectively block the expression of let-7 microRNA family members, which act as tumor suppressors by inhibiting the expression of oncogenes and key regulators of mitogenic pathways, including RAS, MYC, and HMGA2 [2]. Lin28A recruits TUTase to inhibit let-7 precursors to block Dicer processing in the cell cytoplasm, whereas Lin28B represses let-7 maturation through a TUTase-independent mechanism [3]. Many studies indicated that both Lin28A and Lin28B show upregulated expression in human malignancies and that they function as oncogenes by promoting transformation and tumor progression [4, 5].

Overexpression of Lin28A and Lin28B is associated with poor prognosis in various cancers, such as oral squamous cell carcinoma (OSCC) [6, 7], colon cancer [8, 9], epithelial ovarian carcinoma (EOC) [4, 10, 11], gastric cancer [12–14], hepatocellular carcinoma (HCC) [15–17], breast cancer [18, 19], esophagus cancer [20], and other malignancies [21–26]. On basis of these extensive literatures, Lin28A and Lin28B were regarded as promising prognostic factors for multiple cancers. However, the prognostic significance of Lin28A and/or Lin28B varies among different studies. To better confirm its prognostic significance, the present meta-analysis was conducted to evaluate its functional role in predicting cancerous survival of human malignancies.

## Materials and methods

### Search strategy

We carefully performed a comprehensive search on PubMed, Web of Science, and Embase until June 2017 to identify relevant potential literatures. The following set of keywords was simultaneously applied for the online study search: “Lin28” or “Lin28” or “Lin28A” or “Lin28B” and “tumor” or “tumour” or “cancer” or “carcinoma” or “neoplasm” or “malignancies.”

### Inclusion and exclusion criteria

**Fig. 1:**
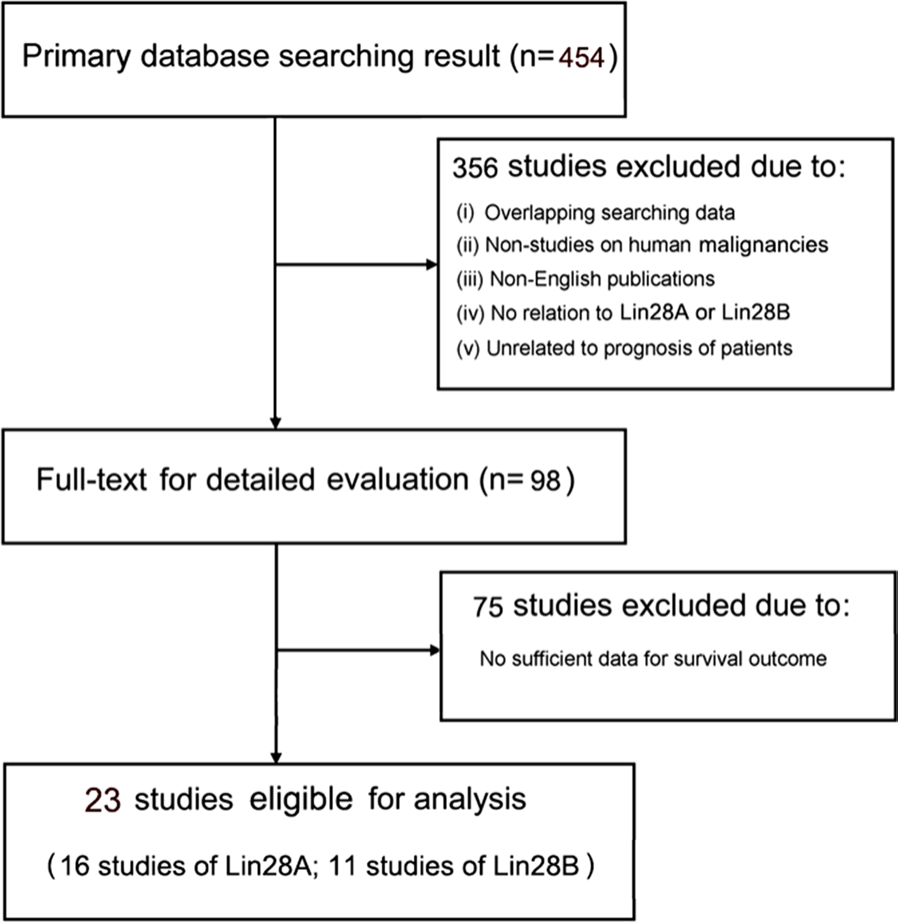
Flow diagram of study selection process

This meta-analysis was strictly conducted in accordance with the guidelines of the preferred reporting items of systematic reviews and meta-analyses (PRISMA) statement [27]. Studies were considered eligible if they met the following criteria: (i) assayed the expression level of Lin28A or Lin28B from human tumor samples; (ii) stratified the expression of Lin28A or Lin28B; and (iii) investigated the association of Lin28A or Lin28B expression levels with cancer overall survival (OS) or progression, along with a corresponding hazard ratio (HR) or survival curves. If more than one article had been published on the same study cohort, only the most comprehensive study was included in this meta-analysis. Moreover, the following criteria were also considered: (i) publications in English, (ii) researches on human malignancies, and (iii) studies on the association of Lin28A or Lin28B expression with cancer prognosis. Furthermore, articles were excluded when they did not cover the points above. Letters, case reports, review articles, and experiments on animals alone were excluded. A flow diagram of the selection process with further details is shown in Fig. 1.

### Data extraction

The extracted data included the following elements: (i) first author and publication year; (ii) characteristics of the studied population, including patients’ nationality, region, mean or median age, study size, disease type, and examined type of sample; (iii) cut-off definition and method of sample analysis; (iv) source of HR, mean, or median follow-up duration; and (v) HRs of elevated Lin28A or Lin28B expression for OS, recurrence-free survival (RFS), disease-free survival (DFS), or progression-free survival (PFS). If HRs were not directly reported, then the survival data were extracted from Kaplan–Meier plots by using Engauge Digitizer V.5.1 (license type: GPL; developed by: Mark Mitch; Category: C:\Science/CAD). Furthermore, HRs with 95% confidence intervals (CIs) were calculated using practical methods by Stata V.12.0 (StataCorp LP, College Station, Texas, USA) [28]. All data above are comprehensively detailed in Tables 1, 2, Additional file 1: Table S1 and Additional file 2: Table S2.

**Table 1:** Main characteristics of studies on Lin28 (Lin28A) and cancer prognosis

First author	Case nationality	Region	Age, years	Included number	Malignant disease	Detected sample	Analysis method	Survival analysis
Hsu, 2015	Chinese Taiwan	Asia	53.80^mean^	140	EOC	Tissue	IHC	PFS
Tu, 2015	USA	North America	NA	595	CRC	Tissue	IHC	OS
Faria, 2015	Germany/Brazil	Europe	< 45.80	124 (DFS)/188 (OS)	ACC	Tissue	IHC	OS/DFS
Qin, 2014	China	Asia	50.00^median^	90	Glioma	Tissue	IHC	OS/PFS
Wang, 2014	China	Asia	59.60^mean^	298	GC	Tissue	IHC	OS/DFS
Liu, 2013	China	Asia	46.00^median^	86	Breast cancer	Tissue	IHC	OS
Xu, 2013	China	Asia	56.00^median^	229	GC	Tissue	IHC	OS
Yin, 2013	China	Asia	49.00^median^	57	HCC	Tissue	qRT-PCR	OS/RFS
Ma, 2013	USA	North America	60.78^mean^	343	EOC	Tissue	AQUA	OS/PFS
Wu, 2013	China	Asia	60.00^mean^	72	OSCC	Tissue	IHC	OS/DFS
Feng, 2012	USA	North America	50.00^median^	569	Breast cancer	Tissue	AQUA	OS
Hamano, 2012	Japan	Asia	65.00^mean^	161	OC	Tissue	IHC	OS/DFS
Qiu, 2012	China	Asia	50.00^median^	53	HCC	Tissue	PCR	OS/RFS
Rodini, 2012	Brazil	South America	NA	37	Medulloblastoma	Tissue	qRT-PCR	OS
Korshunov, 2012	Germany	Europe	< 18.00	816	ETMR	Tissue	IHC	OS
Kim, 2011	South Korea	Asia	48.00^median^	38	NPC	Tissue	IHC	OS/PFS

*EOC* epithelial ovarian cancer, *CRC* colorectal cancer, *ACC* adrenocortical cancer, *GC* gastric cancer, *HCC* hepatocellular carcinoma, *OSCC* oral squamous cell carcinoma, *ETMR* embryonal tumor with multilayered rosettes, *OC* oesophagus cancer, *NPC* nasopharyngeal carcinoma, *PFS* progression-free survival, *OS* overall survival, *DFS* disease-free survival, *RFS* recurrence-free survival, *IHC* immunohistochemistry, *qRT-PCR* quantitative real-time PCR, *AQUA* automated quantitative analysis

**Table 2:** Main characteristics of studies on Lin28B and cancer prognosis

First author	Case nationality	Region	Age, years	Included number	Malignant disease	Detected sample	Analysis method	Survival analysis
Hsu, 2015	Chinese Taiwan	Asia	53.80^mean^	140	EOC	Tissue	IHC	OS/PFS
Tu, 2015	USA	North America	NA	595	CRC	Tissue	IHC	OS
Wang, 2015	China	Asia	46.30^mean^	58	OSCC	Tissue	IHC	OS
Hu, 2014	China	Asia	50.00^mean^	97	GC	Tissue	IHC	OS
Pang, 2014	China	Asia	56.90^mean^	149	Colon cancer	Tissue	IHC	OS/RFS
Cheng, 2013	Chinese Taiwan	Asia	60.00^mean^	96	HCC	Blood	qRT-PCR	RFS
Wu, 2013	China	Asia	60.00^mean^	72	OSCC	Tissue	IHC	OS/DFS
Diskin, 2012	USA	North America	1.42^median^	87	Neuroblastoma	Tissue	qRT-PCR	OS
Hamano, 2012	Japan	Asia	65.00^mean^	137	OC	Tissue	IHC	OS/DFS
King, 2011	USA	North America	66.40^mean^	88	Colon cancer	Tissue	Microarrays	OS/RFS
Lu, 2009	USA	North America	57.90^mean^	211	EOC	Tissue	qRT-PCR	OS/PFS

*EOC* epithelial ovarian cancer, *CRC* colorectal cancer, *GC* gastric cancer, *HCC* hepatocellular carcinoma, *OSCC* oral squamous cell carcinoma, *OC* oesophagus cancer, *PFS* progression-free survival, *OS* overall survival, *DFS* disease-free survival, *RFS* recurrence-free survival, *IHC* immunohistochemistry, *qRT-PCR* quantitative real-time PCR

### Statistical analysis

In this meta-analysis, HRs and corresponding 95% CIs were combined to estimate the prognostic values of high Lin28A or Lin28B expression for cancer prognosis. An individual or pooled HR of more than 1.0 indicated poor prognosis for patients with Lin28A or Lin28B overexpression, and an HR of less than 1.0 represented better prognosis. Moreover, a fixed-effects model or a random-effects model was applied for meta-analysis on the basis of the heterogeneity among the pooled studies [29]. Heterogeneity among studies was evaluated by Chi-square test (assessing the *P* value) and Higgins *I*^2^ statistic. Sensitivity analyses, including influence analysis and Galbraith plot, were implemented for individual studies to identify the source of heterogeneity. If statistically significant heterogeneity was observed (*P *< 0.10 or *I*^2^ > 50%), the random-effects model was applied to estimate the pooled HR; otherwise, the fixed-effects model was used. If necessary, we also classified the included studies into subgroups on the basis of similar characteristics to minimize the influence of significant heterogeneity. In addition, publication bias was estimated using Egger’s test with funnel plot. Stata version 12.0 (Stata Corporation, College Station, TX, USA) was used to calculate all statistical analyses. All *P* values were two sided, and a *P* value of less than 0.05 was considered statistically significant.

## Results

### Characteristics of eligible studies

As shown in Fig. 1, 454 articles related to Lin28A/Lin28B expression in cancers were identified by comprehensive literature search on PubMed, Web of Science, and Embase. Up to 356 studies were excluded by preliminary review, and 75 studies of insufficient survival data (HRs or survival curves) were eliminated by further detailed evaluation. Finally, 23 studies were considered eligible for meta-analysis, 16 of which focused on Lin28A [4, 5, 7, 10, 13, 14, 16–20, 22–26], and 11 studies focused on Lin28B [4–9, 11, 12, 15, 20, 21].

The main characteristics and data of the included studies are summarized in Tables 1, 2. All of 23 eligible studies were retrospective observations in the meta-analysis. In the 16 studies on Lin28A, 3772 cases from Chinese Taiwan, USA, Germany, Brazil, China, Japan, and South Korea were investigated. In the 11 studies on Lin28B, 1730 patients from Chinese Taiwan, USA, China, and Japan were included. All studied populations were classified into different regions, such as Asia, North/South America, and Europe, considering their nationalities. The mean or median age of the included patients ranged from 1.42 to 66.40 years. The malignancies investigated by these studies included OSCC, EOC, HCC, colorectal cancer, adrenocortical cancer, glioma, gastric cancer, breast cancer, esophageal cancer, medulloblastoma, embryonal tumor with multilayered rosettes, nasopharyngeal carcinoma, colon cancer, and neuroblastoma. All the studies detected Lin28A or Lin28B expression on tissue samples, except one study that used peripheral blood [15]. With regard to the main method used to detect Lin28A or Lin28B expression, 14 studies conducted immunohistochemistry, five studies performed quantitative real-time PCR (qRT-PCR), two studies employed automated quantitative analysis (AQUA), one study used PCR, and one study applied microarrays. Up to 13 studies directly reported the values of HR, whereas the HR values of the 10 other studies were extracted from the survival curves. The mean or median length of follow up among these studies ranged from 19.7 to 106.0 months.

### Correlation between Lin28A overexpression and OS

**Fig. 2:**
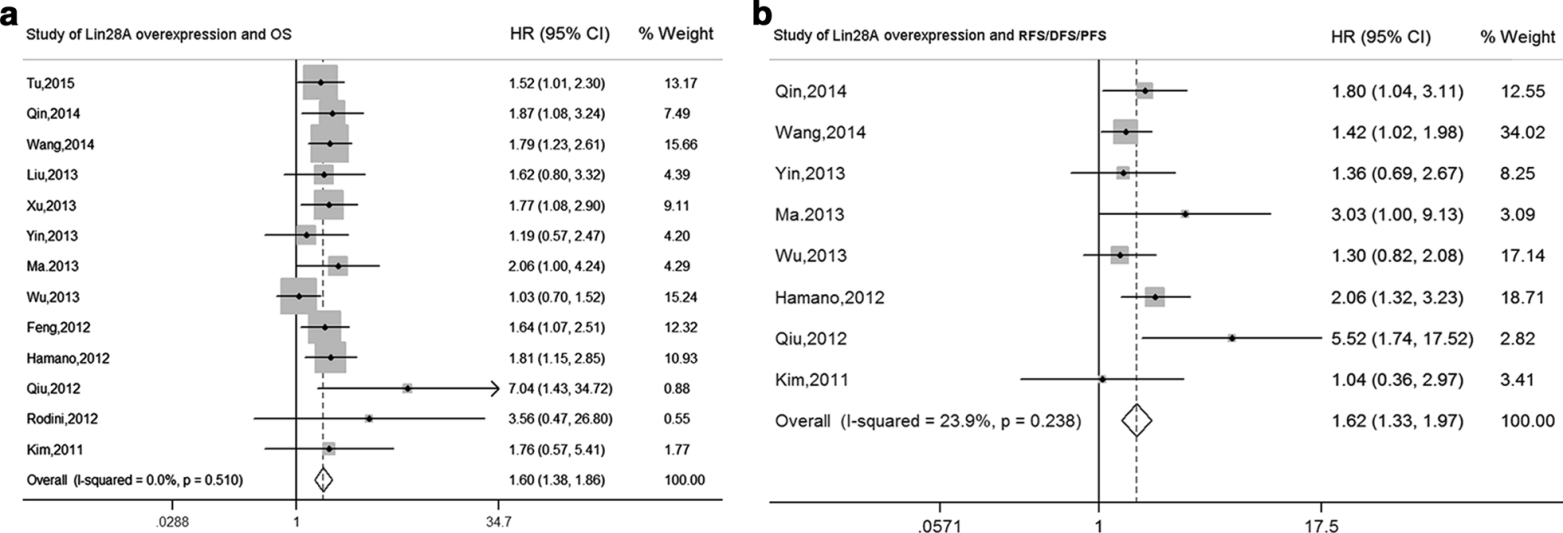
Forest plots summarizing the association of Lin28A overexpression and OS (**a**), and RFS/DFS/PFS (**b**) in patients with various cancers. *HR* hazard ratio, *CI* confidence interval

Statistically significant heterogeneity was found among 15 studies for Lin28A overexpression and OS (*I*^2^= 93.7%, *P *< 0.001) (Additional file 3: Figure S1A). Therefore, influence analysis and Galbraith plot of individual studies were applied for the source of heterogeneity, and the results suggested that two studies of relative low quality (The study of Faria et al. determined the Lin28 protein expression by LIN28 rabbit polyclonal antibody, which recognizes both Lin28A and Lin28B [23], meanwhile the study of Korshunov et al. enrolled ETMR minor patients aged under 18 years [26]) which might lead to the instability and heterogeneity of the pooled outcome (Additional file 4: Figure S2A, B). Meta-analysis and sensitivity analyses were repeated after excluding the two studies (Additional file 4: Figure S2C, D), and the results showed no heterogeneity (*I*^2^= 0.0%, *P *= 0.510). The pooled HR value of Lin28A overexpression for OS was 1.60 (95% CI 1.38, 1.86) (*P *< 0.001), as detected by the fixed-effects model (Fig. 2a). Thus, we considered that the elevated expression level of Lin28A was significantly associated with poor OS in patients with malignancies.

### Correlation between Lin28A overexpression and RFS/DFS/PFS

Statistically significant heterogeneity apparently existed among 10 studies for Lin28A overexpression and RFS/DFS/PFS (*I*^2^= 70.5%, *P *< 0.001) (Additional file 3: Figure S1B). Thus, influence analysis and Galbraith plot for individual studies were also employed (Additional file 5: Figure S3A, B). After eliminating two studies with high heterogeneity [4, 23] (Additional file 5: Figure S3C, D), the outcome of heterogeneity among studies significantly decreased to a low level (*I*^2^= 23.9%, *P *= 0.238). The fixed-effects model was applied, and the pooled HR value of Lin28A overexpression for RFS/DFS/PFS was 1.62 (95% CI 1.33, 1.97) (P < 0.001) (Fig. 2b). Therefore, the results indicated that Lin28A overexpression was significantly related to poor RFS/DFS/PFS for patients.

### Correlation between Lin28B overexpression and OS

**Fig. 3:**
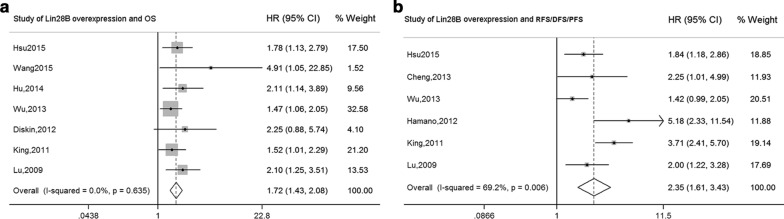
Forest plots summarizing the association of Lin28B overexpression and OS (**a**), and RFS/DFS/PFS (**b**) in patients with various cancers. *HR* hazard ratio, *CI* confidence interval

Among the 10 studies that evaluated the prognostic value of Lin28B overexpression for OS, statistically significant heterogeneity was evident (*I*^2^ = 83.3%, *P *< 0.001) (Additional file 6: Figure S4A). We applied influence analysis and Galbraith plot of individual studies to identify certain studies that might result in the instability of the pooled outcome (Additional file 7: Figure S5). No heterogeneity remained when three studies [5, 8, 20] were excluded (*I*^2^ = 0.00%, *P *= 0.635), and the pooled HR detected by the fixed-effects model demonstrated that high Lin28B expression significantly correlated with poor OS of human malignancies (HR = 1.72, 95% CI 1.43, 2.08) (P < 0.001) (Fig. 3a).

### Correlation between Lin28B overexpression and RFS/DFS/PFS

Significant heterogeneity was evident for the seven studies on Lin28B overexpression and RFS/DFS/PFS (*I*^2^ = 83.2%, *P *< 0.001) (Additional file 6: Figure S4B), and one low-quality study (No detailed information was shown about the anti-body for IHC, and no description of the case number for high Lin28B expression, and for low Lin28B expression was reported [8]) was excluded by influence analysis and Galbraith plot (Additional file 8: Figure S6). The pooled HR of Lin28B overexpression for RFS/DFS/PFS was 2.35 (95% CI 1.61, 3.43), as detected by the random-effects model, and significant heterogeneity was retained among the studies (*I*^2^= 69.2%, *P *= 0.006) (Fig. 3b). Therefore, subgroup analyses were correspondingly performed on the basis of similar characteristics, such as nationality, region, and malignant disease. No heterogeneity was found in the subgroups of Chinese Taiwan (*I*^2^ = 0.0%, *P *= 0.667) and EOC (*I*^2^ = 0.0%, *P *= 0.806) (Additional file 9: Figure S7). By contrast, significant heterogeneity was found in the subgroups of Asia (*I*^2^ = 64.9%, *P *= 0.036) and North America (*I*^2^ = 70.6%, *P *= 0.065) (Additional file 10: Figure S8).

### Publication bias

**Fig. 4:**
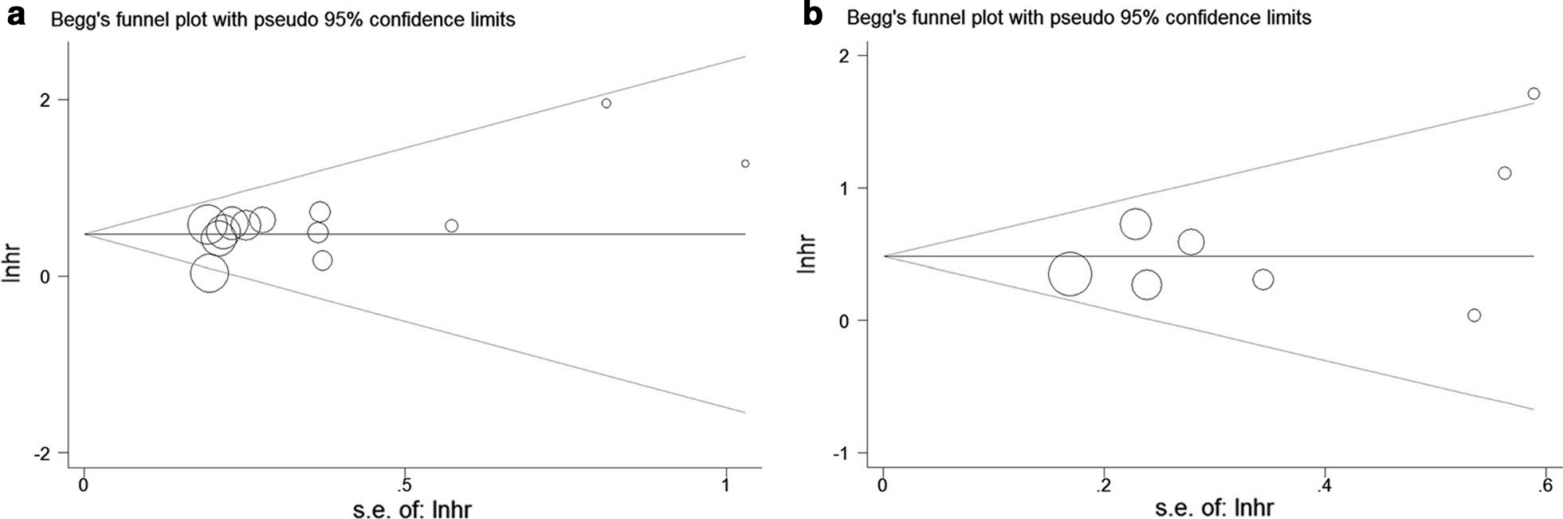
Begg’s funnel plots used to assess publication bias. **a** Funnel plot relating to analysis of Lin28A overexpression and OS. **b** Funnel plot relating to analysis of Lin28A overexpression and RFS/DFS/PFS

Egger’s test with funnel plots were used to evaluate the publication bias among the eligible studies. In the pooled analyses of studies on Lin28A expression level with OS and RFS/DFS/PFS, the *P* values for Egger’s test were 0.069 and 0.239. Begg’s test reached *P* values of 0.127 and 0.266, respectively, suggesting no significant publication bias (Fig. 4a, b). Because there were less than 10 studies in the pooled analyses of Lin28B expression level with OS and RFS/DFS/PFS, we did not assess the publication bias according to Cochrane Guidelines [30].

## Discussion

Lin28A and its homolog Lin28B belong to highly conserved RNA-binding proteins family, which are found to involve in numerous biological processes, including cell development, pluripotency, reprogramming, and oncogenesis [31, 32]. Several recent investigations have confirmed that Lin28A and Lin28B can regulate gene expression either by directly binding to messenger RNAs (mRNAs) or by blocking microRNA biogenesis, and the underlying mechanisms include Let-7-family-dependent and Let-7-family-independent modes of action [3].

Let-7 and lin28 (Lin28A and Lin28B) were first identified through mutagenesis screening as heterochronic genes in *Caenorhabditis elegans* [33, 34], and the expression and regulation of lin28 and let-7 are highly conserved throughout evolution [35]. Recent studies have established the lin28/let-7 pathway as a central regulator of mammalian glucose metabolism [36]. Although the exact roles of the let-7 family in adult mammalian tissues have not been definitely characterized, let-7 is known to serve as a tumor suppressor. Considerable evidence demonstrated that let-7 expression level is downregulated in various cancers and that let-7 overexpression restrains the growth and metastatic potential of cancer cells [37–40]. As one targeting gene of let-7, lin28 expression is upregulated in various tumors, such as OSCC [6, 7], colon cancer [8, 9], EOC [4, 10, 11], gastric cancer [12–14], HCC [15–17], and breast cancer [18, 19]. Although many major effects of lin28 are mediated by blocking let-7 microRNA biogenesis, lin28 can also directly bind to GGAGA (G, guanosine; A, adenosine) sequences enriched with loop structures in mRNA targets, and this activity is similar to its interaction with let-7 microRNA precursors [41]. Many of these targets function as oncogenes or tumor growth indicators, such as insulin-like growth factor 2 (IGF-2), a crucial growth and differentiation factor for muscle tissues [42], and IGF-2 mRNA-binding protein 2, which binds several mRNAs encoding mitochondrial respiratory chain complex subunits [43]. Therefore, Lin28A and Lin28B, along with their target genes, deserve further analysis in the future.

In this meta-analysis, we investigated the prognostic values of Lin28A and Lin28B for multiple human malignancies. Combining the outcomes of studies regarding the association of lin28 expression and tumor prognosis, we have successfully drawn many valuable results. First, increased expression level of Lin28A was considered to predict poor OS and RFS/DFS/PFS for cancer patients, with combined HR values of 1.60 (95% CI 1.38, 1.86) and 1.62 (95% CI 1.33, 1.97), respectively. Second, considering the pooled outcomes of studies on the relation between Lin28B expression and cancer prognosis, we found that elevated Lin28B level was significantly associated with poor OS and RFS/DFS/PFS of malignant diseases, with pooled HR values of 1.72 (95% CI 1.43, 2.08) and 2.35 (95% CI 1.61, 3.43), correspondingly, which also exerted statistical significance. Our findings suggested that Lin28A and Lin28B are promising biomarkers, and the detection of Lin28A and Lin28B expression in cancer patients is of potential value for monitoring patients’ survival.

However, significant heterogeneity was observed in the initial meta-analysis (Additional file 3: Figure S1 and Additional file 6: Figure S4). Therefore, influence analysis and Galbraith plot were applied for individual studies to investigate the source of heterogeneity. After excluding several studies of relatively low quality, no heterogeneity was found among the studies on cancer prognosis and lin28 expression, except for those concerning the association between Lin28B expression and RFS/DFS/PFS. Furthermore, subgroup analyses were performed to minimize the influence of heterogeneity (*I*^2^ = 69.2%, *P *= 0.006). In subgroup analyses, no heterogeneity was observed in the subtotals of Chinese Taiwan (*I*^2^ = 0.0%, *P *= 0.667) and EOC (*I*^2^ = 0.0%, *P *= 0.806). Also, no heterogeneity was shown in the subgroup analyses by different assay methods for Lin28A overexpression and OS, and RFS/DFS/PFS (Additional file 11: Figure S9A, B), and for Lin28B overexpression and OS (Additional file 11: Figure S9C). However, the subgroup of IHC still indicated of high heterogeneity for Lin28B overexpression and RFS/DFS/PFS (*I*^2^ = 75.8%, *P *= 0.016) (Additional file 11: Figure S9D). Besides, the subgroups of Asia (*I*^2^ = 64.9%, *P *= 0.036) and North America (*I*^2^ = 70.6%, *P *= 0.065) showed significant heterogeneity (Additional file 10: Figure S8). Therefore, we consider that the source of heterogeneity might result from the influence of different populations and disease types of the patients, rather than different methods.

Despite the meta-analysis was performed with rigorous statistics, our conclusion still has several limitations for the following reasons. First, the amounts of included studies in the meta-analysis was not sufficiently enough for more powerful results, as well as the study numbers for each cancer type. Second, all eligible studies were retrospective for analysis, which might impair the credibility of meta-analysis. Third, the population diversity, disease type, source of tissue sample and antibody for IHC may cause heterogeneity to a certain extent. Moreover, many researchers used a median immunohistochemical score as a division value, but the median scores slightly differed among studies. Several studies also performed a ternary method to classify the expression levels of Lin28A into high, medium, and low categories, which might result in an undervalued HR for Lin28A [19]. All the disadvantages above might cause heterogeneity in the meta-analysis and produce deviation when evaluating the prognostic significances of Lin28A and Lin28B in human malignancies. Also, HR values of several studies were calculated using data extracted from the survival curves, which might unavoidably cause slight statistical errors.

## Conclusion

To summarize, Lin28A and Lin28B provide significant prognostic values in various human malignancies. Overexpression of Lin28A or Lin28B suggests poor prognosis for cancer patients. In consideration of the complicated regulatory mechanism between lin28 and its target genes, further investigation and additional relevant studies are required to establish the clinical significance of Lin28A and Lin28B as ideal prognostic biomarkers.

## Additional files

**Additional file 1: Table S1.** Main characteristics of studies on Lin28 (Lin28A) and cancer prognosis.

**Additional file 2: Table S2.** Main characteristics of studies on Lin28B and cancer prognosis.

**Additional file 3: Figure S1.** In initial meta-analysis, forest plots summarizing the association of Lin28A overexpression and OS (A), and RFS/DFS/PFS (B) in patients with various cancers.

**Additional file 4: Figure S2.** Influence analysis and Galbraith plot of individual studies on Lin28A expression and OS. A, B Influence analysis and Galbraith plot for initial meta-analysis; C, D influence analysis and Galbraith plot after study exclusion.

**Additional file 5: Figure S3.** Influence analysis and Galbraith plot of individual studies on Lin28A expression and RFS/DFS/PFS. A, B Influence analysis and Galbraith plot for initial meta-analysis; C, D influence analysis and galbraith plot after study exclusion.

**Additional file 6: Figure S4.** In initial meta-analysis, forest plots summarizing the association of Lin28B overexpression and OS (A), and RFS/DFS/PFS (B) in patients with various cancers.

**Additional file 7: Figure S5.** Influence analysis and Galbraith plot of individual studies on Lin28B expression and OS. A, B Influence analysis and Galbraith plot for initial meta-analysis; C, D influence analysis and Galbraith plot after study exclusion.

**Additional file 8: Figure S6.** Influence analysis and Galbraith plot of individual studies on Lin28B expression and RFS/DFS/PFS. A, B Influence analysis and Galbraith plot for initial meta-analysis; C, D influence analysis and Galbraith plot after study exclusion.

**Additional file 9: Figure S7.** Forest plots evaluating the association of Lin28B overexpression and RFS/DFS/PFS in subgroups of Chinese Taiwan and EOC. HR, hazard ratio; CI, confidence interval; EOC, epithelial ovarian carcinoma.

**Additional file 10: Figure S8.** Forest plots evaluating the association of Lin28B overexpression and RFS/DFS/PFS in subgroups of Asian and North America.

**Additional file 11: Figure S9.** Subgroup analyses by different assay methods evaluating the association of (A) Lin28A overexpression and OS, (B) Lin28A overexpression and RFS/DFS/PFS, (C) Lin28B overexpression and OS, (D) Lin28B overexpression and RFS/DFS/PFS.

## Abbreviations

HR: hazard ratio
CI: confidence interval
OS: overall survival
RFS: recurrence-free survival
DFS: disease-free survival
PFS: progression-free survival
OSCC: oral squamous cell carcinoma
EOC: epithelial ovarian carcinoma
HCC: hepatocellular carcinoma
