# Supplementary material for: Prognostic value of Lin28A and Lin28B in various human malignancies: a systematic review and meta-analysis
Source: Cancer Cell Int. 2019 Apr 2;19:79. doi: 10.1186/s12935-019-0788-z (PMC6444518; doi:10.1186/s12935-019-0788-z)
Supplement: Supplementary file 1 — Additional file 1: Table S1. Main characteristics of studies on Lin28 (Lin28A) and cancer prognosis. [file 12935_2019_788_MOESM1_ESM.docx]

**Table S1.** Main characteristics of studies on Lin28 (Lin28A) and cancer prognosis

|  | |  |  |  | **HR (95% CI)** | |
| --- | --- | --- | --- | --- | --- | --- |
| **First author** | **Cut-off value** | | **Source of HR** | **follow-up, month** | **OS** | **RFS/DFS/PFS** |
| Hsu, 2015 | Score | | Reported | 39.0^median^ | NA | 0.760 (0.480, 1.180) |
| Tu, 2015 | Score | | SC | NA | 1.524 (1.009, 2.301) | NA |
| Faria, 2015 | Score | | Reported | NA | 0.710 (0.650, 0.790) | 0.440 (0.230, 0.830) |
| Qin, 2014 | High (++ and +++)/low (+ and -) | | Reproted | NA | 1.874 (1.085, 3.237) | 1.798 (1.040, 3.110) |
| Wang, 2014 | Positive and Negative | | SC | 35.0^mean^ | 1.790 (1.230, 2.620) | 1.417 (1.016, 1.976) |
| Liu, 2013 | Positive and Negative | | SC | 57.0^median^ | 1.624 (0.795, 3.317) | NA |
| Xu, 2013 | Score ≥2 | | Reported | 42.4^median^ | 1.768 (1.077, 2.903) | NA |
| Yin, 2013 | X-tile program, X=4.2 | | SC | 22.0^median^ | 1.188 (0.572, 2.464) | 1.360 (0.692, 2.672) |
| Ma, 2013 | 668.16^mean^ | | Reported | NA | 2.058 (1.000, 4.239) | 3.027 (1.004, 9.127) |
| Wu, 2013 | 6^median^ | | Reported | NA | 1.033 (0.704, 1.515) | 1.304 (0.816, 2.083) |
| Feng, 2012 | <3098, 3098~4446, >4446 | | Reported | 106.0^median^ | 1.640 (1.070, 2.510) | NA |
| Hamano, 2012 | Score | | SC | 32.7^median^ | 1.810 (1.151, 2.845) | 2.064 (1.318, 3.232) |
| Qiu, 2012 | Positive and Negative | | Reproted | 31.0^median^ | 7.039 (1.427, 34.724) | 5.518 (1.738, 17.520) |
| Rodini, 2012 | Five-fold | | SC | NA | 3.561 (0.473, 26.801) | NA |
| Korshunov, 2012 | Positive and Negative | | SC | NA | 10.048 (6.756, 14.944) | NA |
| Kim, 2011 | Positive and Negative | | Reproted | 30.0^median^ | 1.760 (0.570, 5.390) | 1.040 (0.360, 2.940) |

HR, hazard ratio; CI, confidence intervals; SC, data extracted from survival curve; OS, overall survival; DFS, disease-free survival; RFS, recurrence-free survival; PFS, progression-free survival.
