# Supplementary material for: Prognostic value of Lin28A and Lin28B in various human malignancies: a systematic review and meta-analysis
Source: Cancer Cell Int. 2019 Apr 2;19:79. doi: 10.1186/s12935-019-0788-z (PMC6444518; doi:10.1186/s12935-019-0788-z)
Supplement: Supplementary file 2 — Additional file 2: Table S2. Main characteristics of studies on Lin28B and cancer prognosis. [file 12935_2019_788_MOESM2_ESM.docx]

**Table S2.** Main characteristics of studies on Lin28B and cancer prognosis

|  |  |  |  | **HR (95% CI)** | |
| --- | --- | --- | --- | --- | --- |
| **First author** | **Cut-off value** | **Source of HR** | **follow-up, month** | **OS** | **RFS/DFS/PFS** |
| Hsu, 2015 | Score | Reported | 39^median^ | 1.776 (1.129, 2.793) | 1.840 (1.180, 2.860) |
| Tu, 2015 | Score | SC | NA | 1.192 (0.884, 1.607) | NA |
| Wang, 2015 | Score | Reported | 27.4^mean^ | 4.905 (1.053, 22.846) | NA |
| Hu, 2014 | 4.5^median^ | Reported | NA | 2.108 (1.142, 3.889) | NA |
| Pang, 2014 | Score | SC | NA | 20.351 (9.023, 45.898) | 14.203 (6.303, 32.005) |
| Cheng, 2013 | ≤38CT | Reported | 19.7^median^ | NA | 2.248 (1.012, 4.995) |
| Wu, 2013 | 4.5^median^ | Reported | NA | 1.473 (1.057, 2.053) | 1.423 (0.986, 2.052) |
| Diskin, 2012 | NA | SC | NA | 2.253 (0.884, 5.740) | NA |
| Hamano, 2012 | Score | SC | 32.7^median^ | 3.896 (2.277, 6.667) | 5.181 (2.315, 11.494) |
| King, 2011 | Score | SC | NA | 1.520 (1.010, 2.300) | 3.708 (2.413, 5.697) |
| Lu, 2009 | Median | Reported | 31^median^ | 2.100 (1.260, 3.530) | 2.000 (1.220, 3.290) |

HR, hazard ratio; CI, confidence intervals; SC, data extracted from survival curve; OS, overall survival; DFS, disease-free survival; RFS, recurrence-free survival; PFS, progression-free survival.
