# Supplementary figures and images for: Prognostic value of Lin28A and Lin28B in various human malignancies: a systematic review and meta-analysis
Source: Cancer Cell Int. 2019 Apr 2;19:79. doi: 10.1186/s12935-019-0788-z (PMC6444518; doi:10.1186/s12935-019-0788-z)

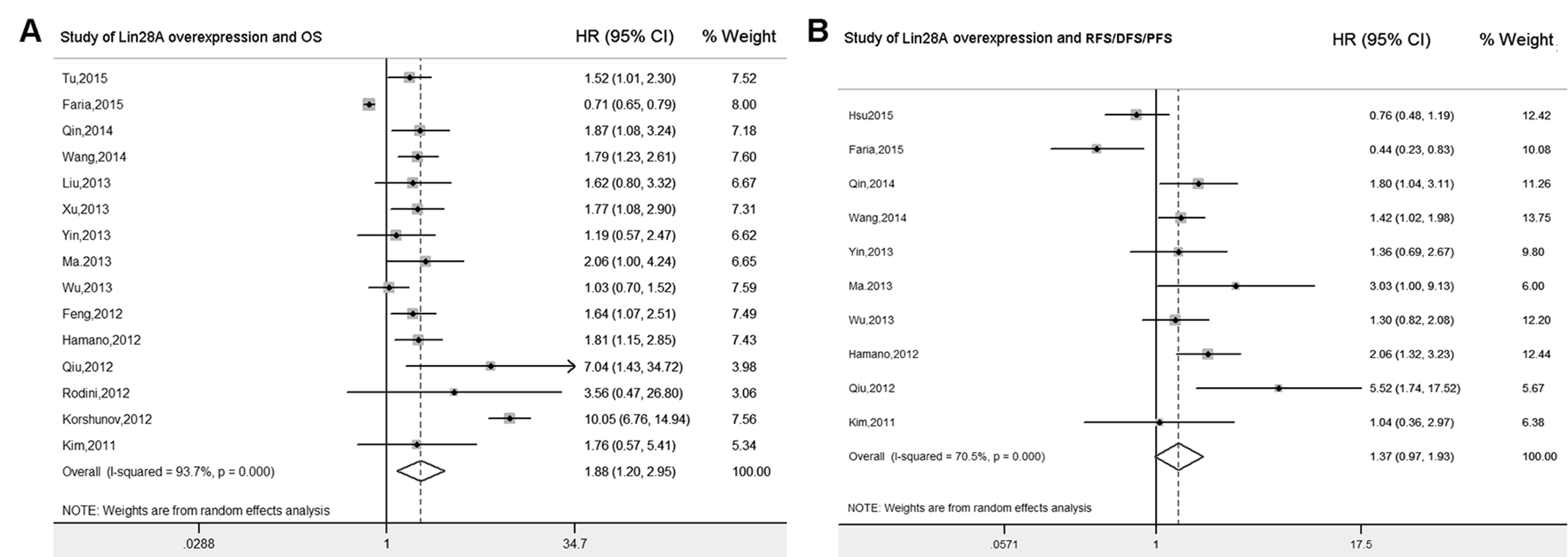

Supplement: Supplementary file 3 — Additional file 3: Figure S1. In initial meta-analysis, forest plots summarizing the association of Lin28A overexpression and OS (A), and RFS/DFS/PFS (B) in patients with various cancers. [file 12935_2019_788_MOESM3_ESM.tif]

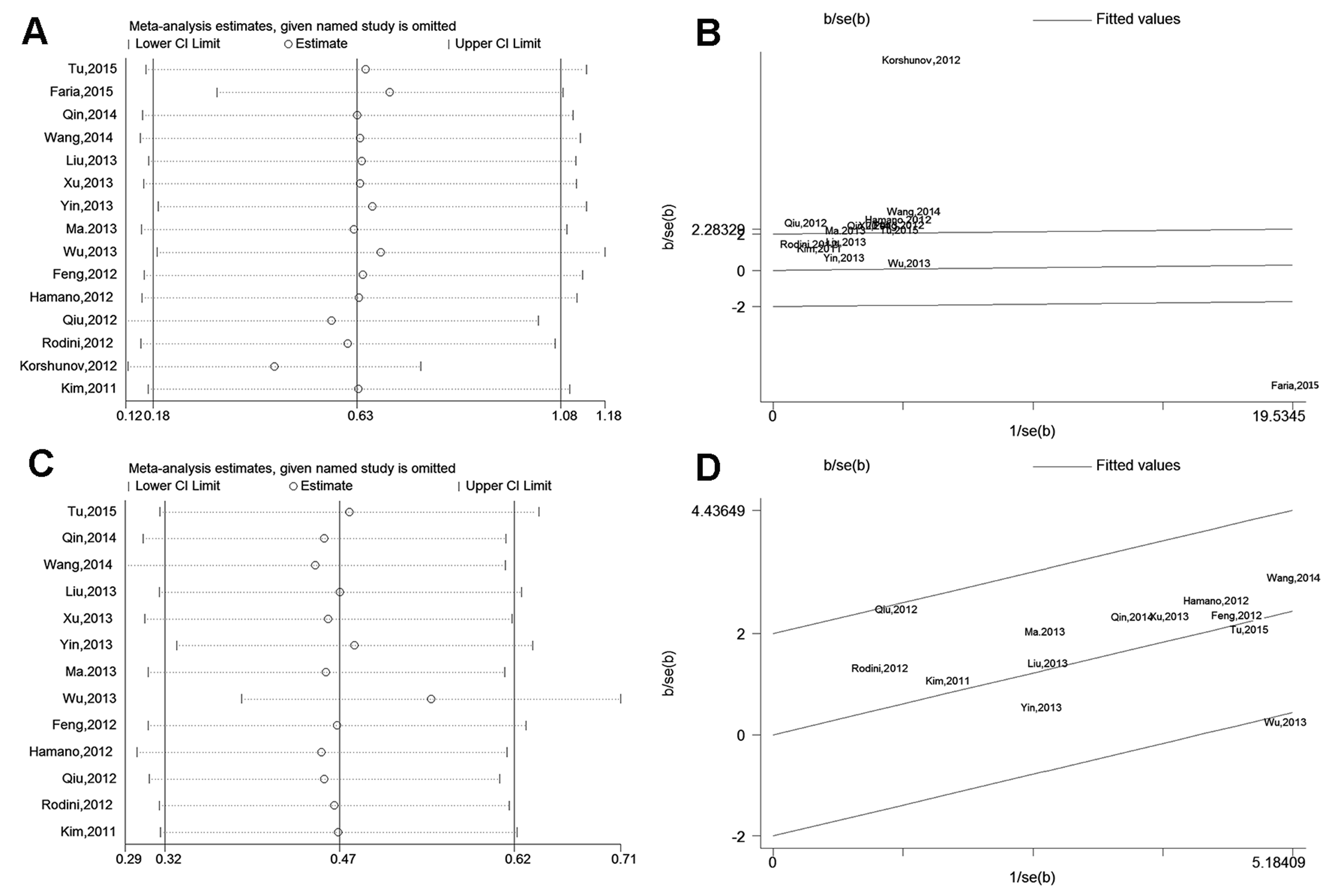

Supplement: Supplementary file 4 — Additional file 4: Figure S2. Influence analysis and Galbraith plot of individual studies on Lin28A expression and OS. A, B Influence analysis and Galbraith plot for initial meta-analysis; C, D influence analysis and Galbraith plot after study exclusion. [file 12935_2019_788_MOESM4_ESM.tif]

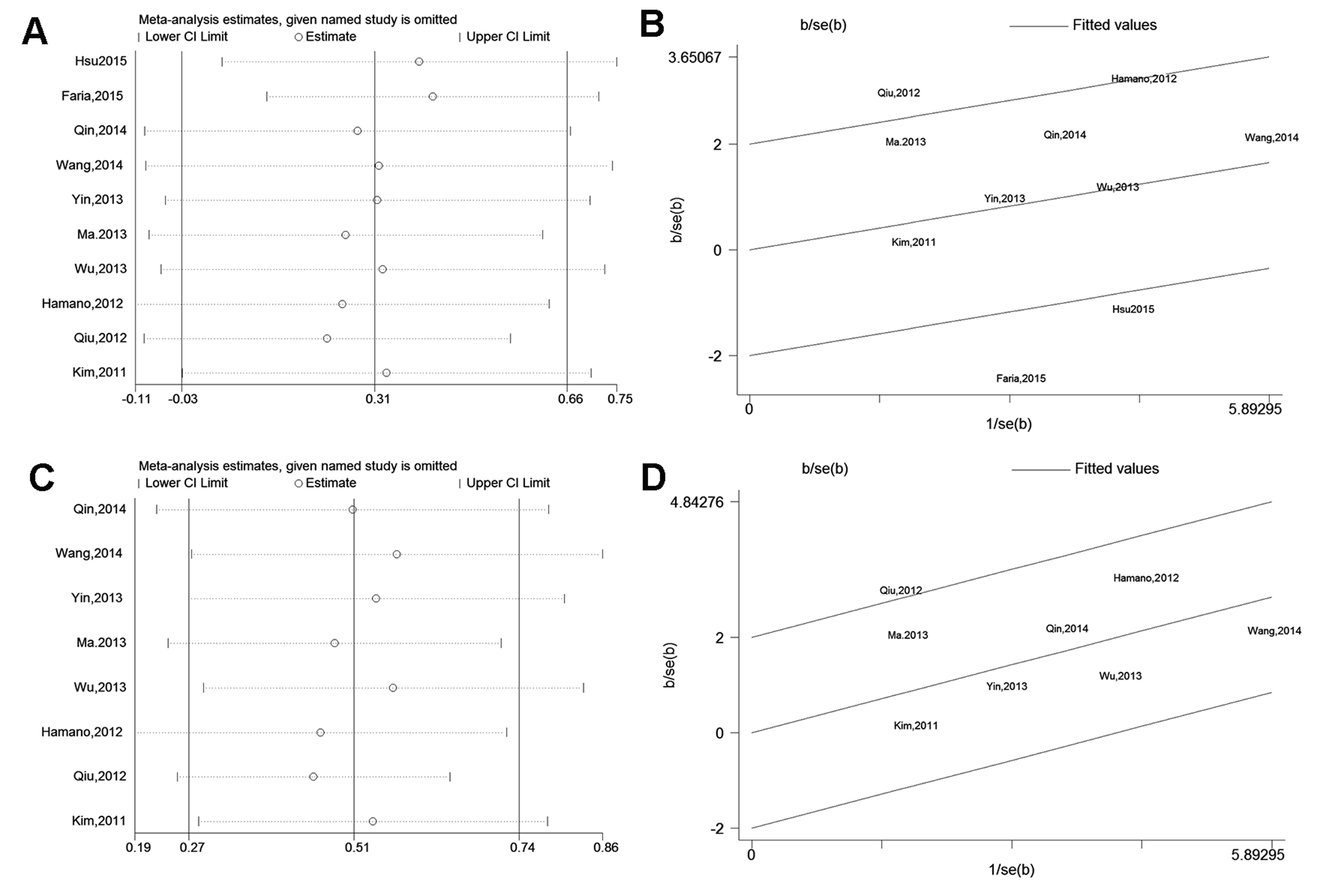

Supplement: Supplementary file 5 — Additional file 5: Figure S3. Influence analysis and Galbraith plot of individual studies on Lin28A expression and RFS/DFS/PFS. A, B Influence analysis and Galbraith plot for initial meta-analysis; C, D influence analysis and galbraith plot after study exclusion. [file 12935_2019_788_MOESM5_ESM.tif]

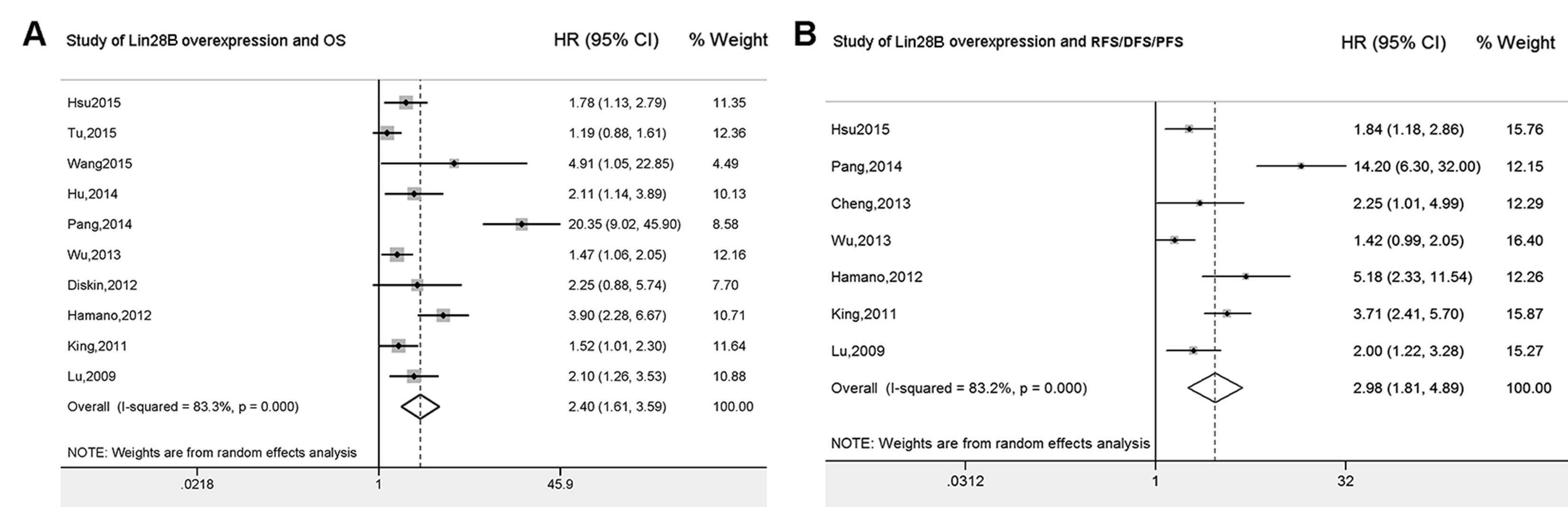

Supplement: Supplementary file 6 — Additional file 6: Figure S4. In initial meta-analysis, forest plots summarizing the association of Lin28B overexpression and OS (A), and RFS/DFS/PFS (B) in patients with various cancers. [file 12935_2019_788_MOESM6_ESM.tif]

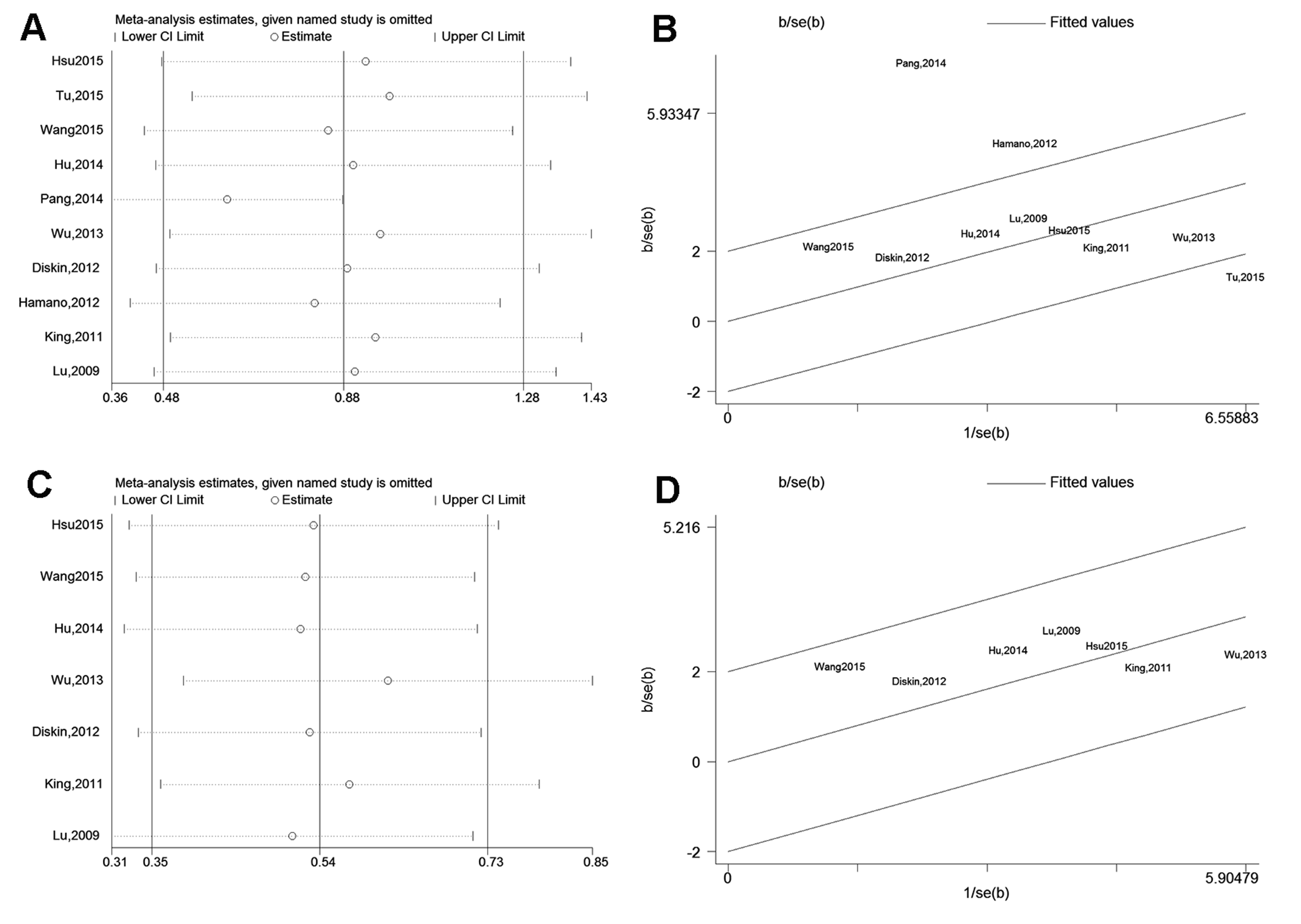

Supplement: Supplementary file 7 — Additional file 7: Figure S5. Influence analysis and Galbraith plot of individual studies on Lin28B expression and OS. A, B Influence analysis and Galbraith plot for initial meta-analysis; C, D influence analysis and Galbraith plot after study exclusion. [file 12935_2019_788_MOESM7_ESM.tif]

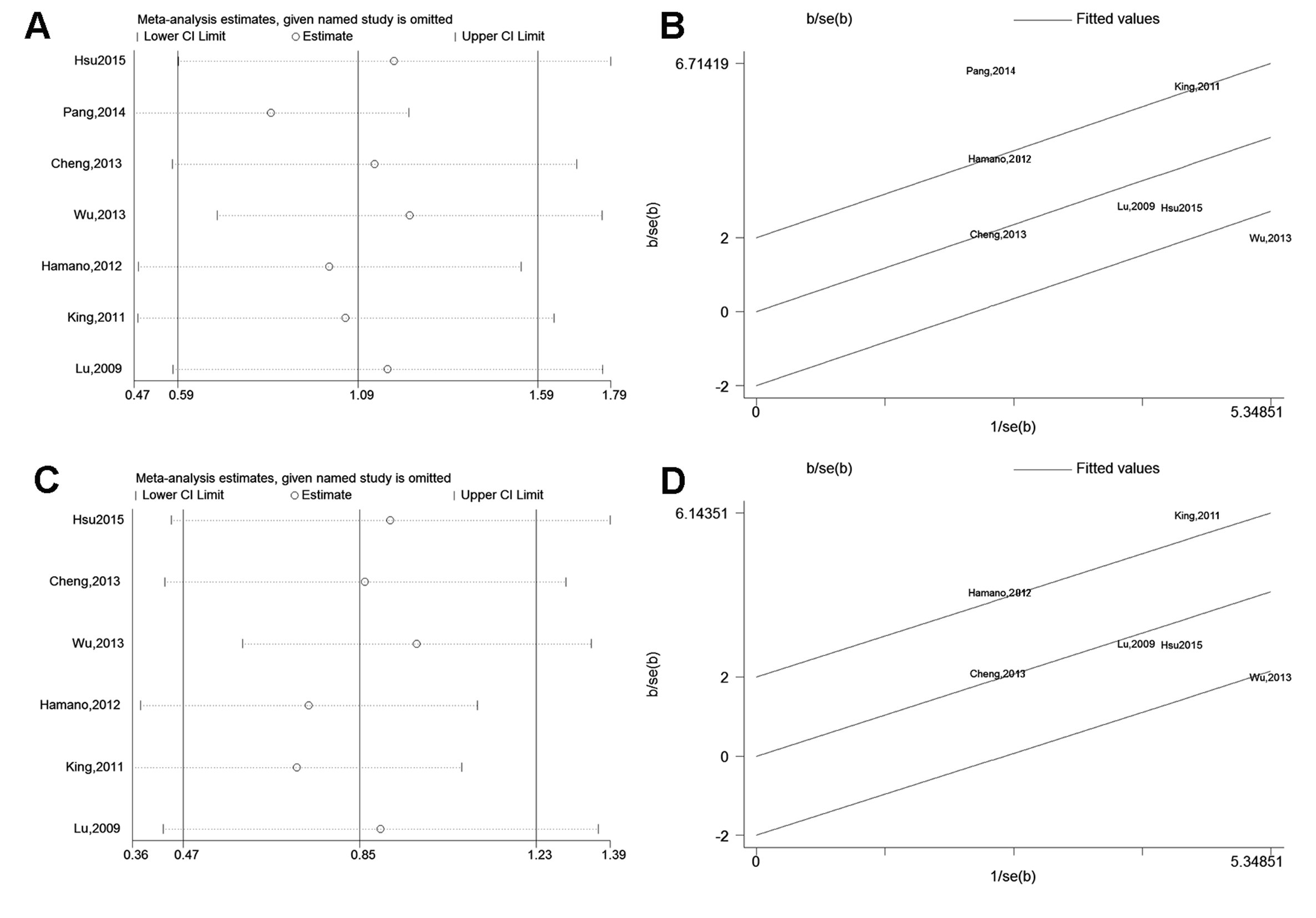

Supplement: Supplementary file 8 — Additional file 8: Figure S6. Influence analysis and Galbraith plot of individual studies on Lin28B expression and RFS/DFS/PFS. A, B Influence analysis and Galbraith plot for initial meta-analysis; C, D influence analysis and Galbraith plot after study exclusion. [file 12935_2019_788_MOESM8_ESM.tif]

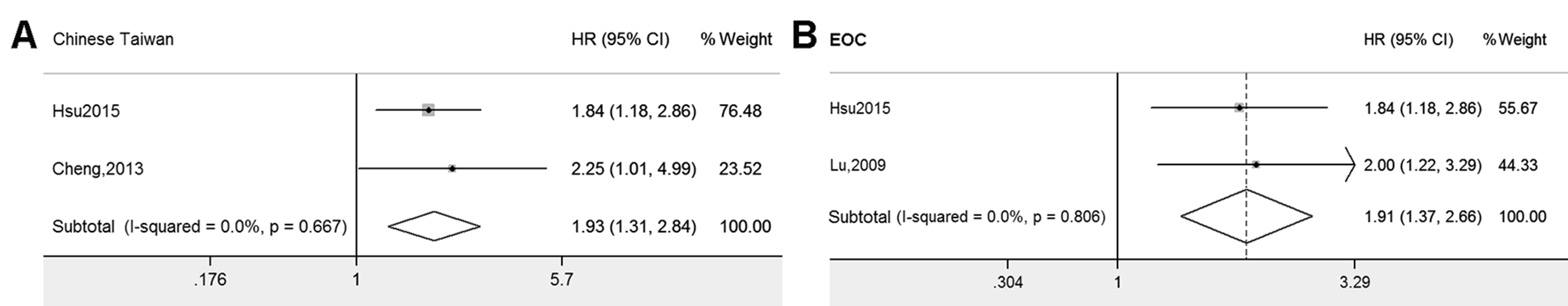

Supplement: Supplementary file 9 — Additional file 9: Figure S7. Forest plots evaluating the association of Lin28B overexpression and RFS/DFS/PFS in subgroups of Chinese Taiwan and EOC. HR, hazard ratio; CI, confidence interval; EOC, epithelial ovarian carcinoma. [file 12935_2019_788_MOESM9_ESM.tif]

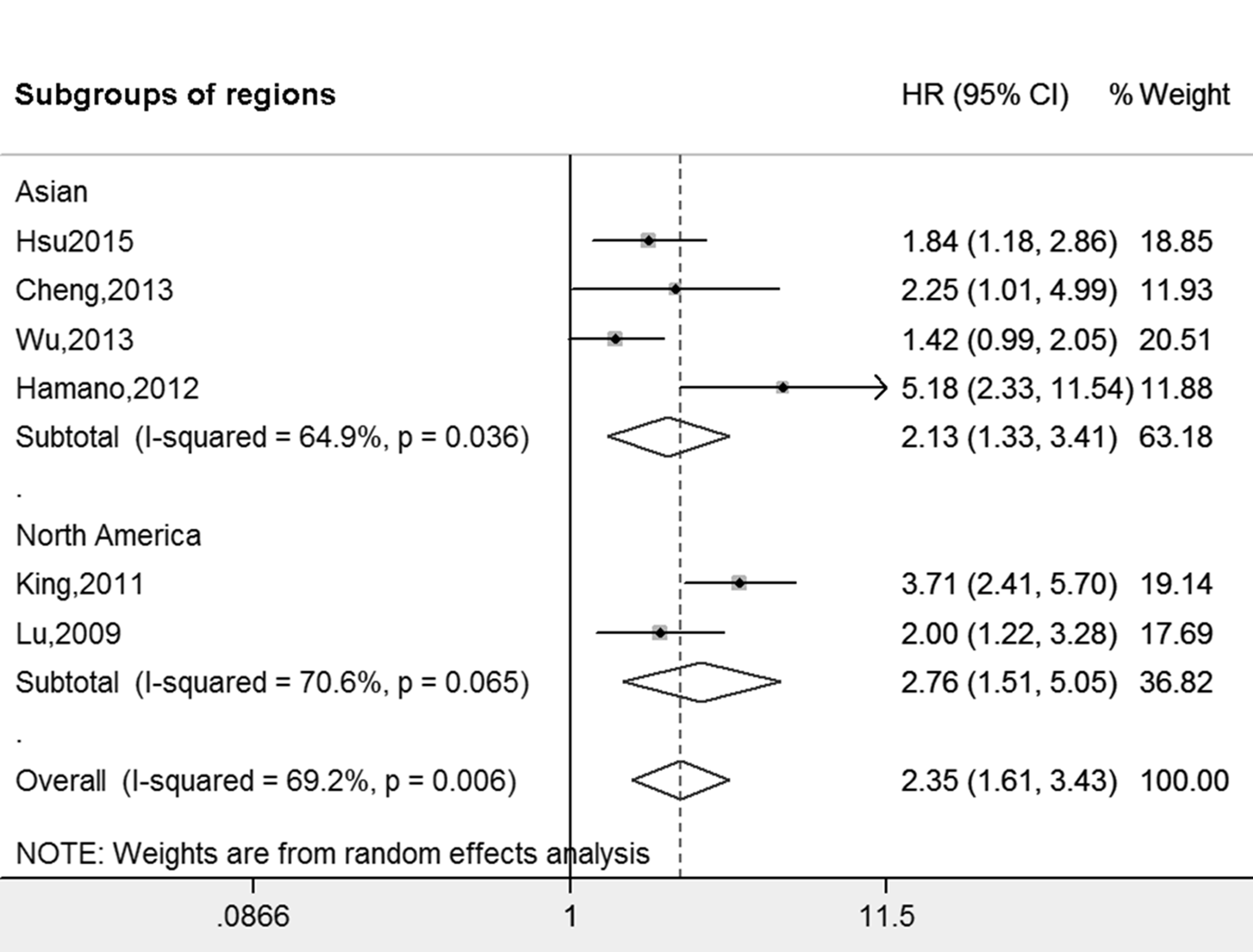

Supplement: Supplementary file 10 — Additional file 10: Figure S8. Forest plots evaluating the association of Lin28B overexpression and RFS/DFS/PFS in subgroups of Asian and North America. [file 12935_2019_788_MOESM10_ESM.tif]

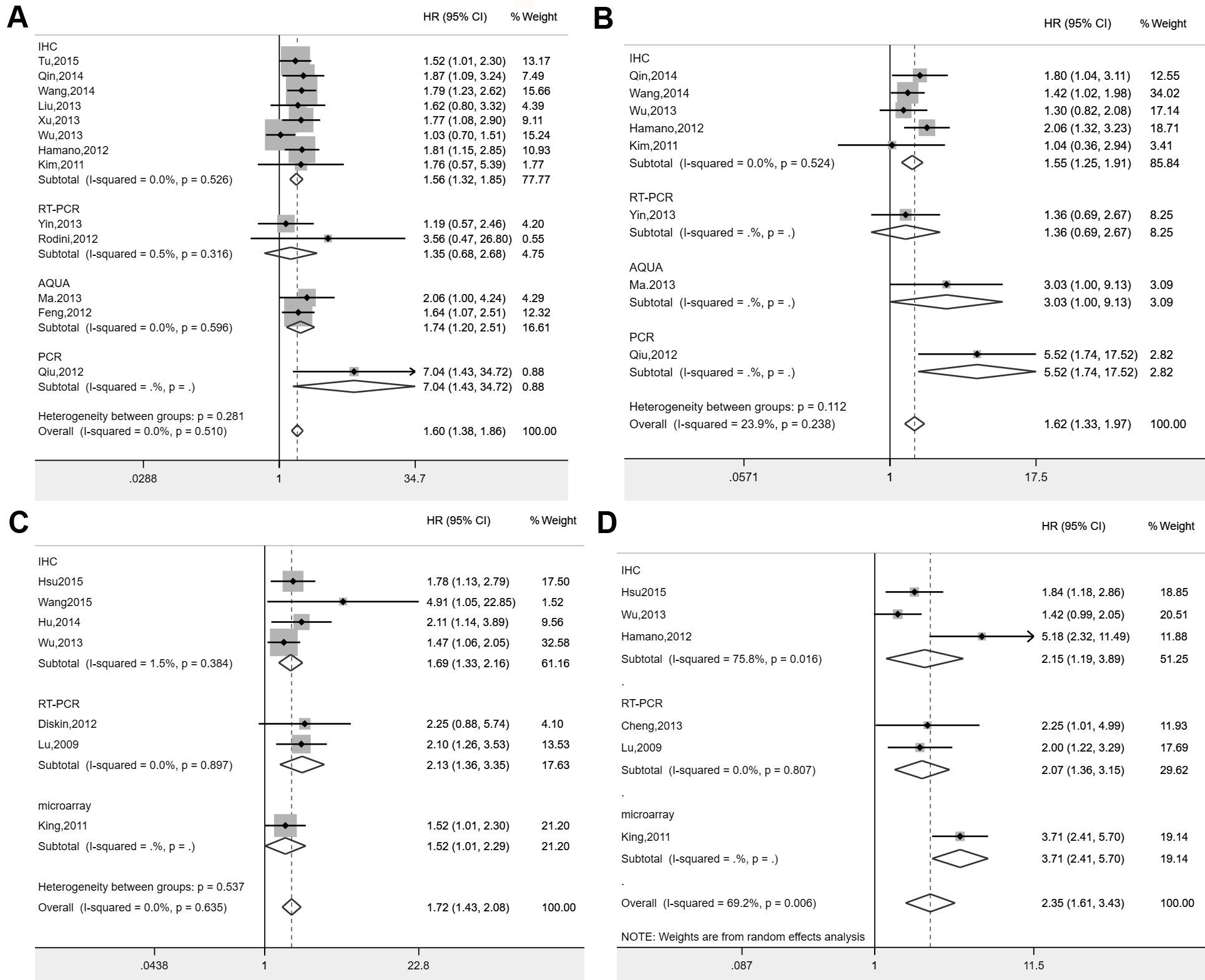

Supplement: Supplementary file 11 — Additional file 11: Figure S9. Subgroup analyses by different assay methods evaluating the association of (A) Lin28A overexpression and OS, (B) Lin28A overexpression and RFS/DFS/PFS, (C) Lin28B overexpression and OS, (D) Lin28B overexpression and RFS/DFS/PFS. [file 12935_2019_788_MOESM11_ESM.tif]
